# Current and Emerging Targeted Therapy in Advanced Gastroesophageal Adenocarcinoma

**DOI:** 10.3390/ph19091420

**Published:** 2026-09-08

**Authors:** Oliver Oakley, Umair Mahmood, Yusuf Ahmad, Elizabeth Smyth

**Affiliations:** 1 Department of Oncology, Oxford University Hospitals NHS Foundation Trust, Oxford OX3 7LJ, UK; oliver.oakley1@ouh.nhs.uk (O.O.); umair.mahmood@ouh.nhs.uk (U.M.); 2 Hilltops Medical Centre, Kensington Dr, Great Holm, Milton Keynes MK8 9HN, UK; yusuf.ahmad4@nhs.net

**Keywords:** advanced gastroesophageal adenocarcinoma, HER2, CLDN18.2, VEGFR, targeted therapy, bispecific antibodies

## Abstract

**Background/Objectives**: Advanced gastroesophageal adenocarcinoma (GEA) carries a poor prognosis, with median overall survival of 13–20 months despite standard chemotherapy. Since trastuzumab’s approval, biomarker-driven precision therapies have expanded rapidly. This review comprehensively summarises current and emerging targeted agents across the key molecular targets driving advanced GEA. **Methods**: A comprehensive literature review was conducted using PubMed, Google Scholar and Cochrane Library in accordance with PRISMA guidelines, searching English-language human studies published between February and July 2026, supplemented by updates during editing to reflect current standards. **Results**: HER2-directed therapy has progressed from trastuzumab through dual blockade, immunotherapy combinations, next-generation ADCs (notably trastuzumab deruxtecan) and bispecific antibodies such as zanidatamab, which has now overtaken trastuzumab in the first-line setting. CLDN18.2-targeted zolbetuximab has demonstrated survival benefit in biomarker-selected patients, with newer ADCs, BiTEs and the first approved solid-tumour CAR-T therapy (satricabtagene autoleucel) extending this target further. VEGFR2 inhibition with ramucirumab remains a cornerstone in later lines, while novel VEGF/PD-1(L1) bispecifics are under investigation. FGFR2b-targeted bemarituzumab showed early promise that weakened on phase 3 confirmation, and MET/EGFR-directed agents, including savolitinib and amivantamab, require stringent biomarker selection to demonstrate benefit amid tumour heterogeneity. **Conclusions**: Novel targeted agents, particularly to HER2 and CLDN18.2, have demonstrated survival benefit despite ongoing challenges. Other lines are more investigational.

## 1. Introduction

Gastric cancer is the fifth most commonly diagnosed cancer worldwide, with approximately 968,000 new cases estimated in 2022 [1]. Chronic *Helicobacter pylori* infection, smoking, alcohol consumption, high salt diet, elevated body mass index and Epstein–Barr virus have all been implicated in its pathogenesis. Worryingly, the incidence of early-onset gastric cancer is rising [2]. Gastroesophageal adenocarcinoma (GEA) encompasses gastric (G), gastroesophageal junction (GOJ) and oesophageal adenocarcinomas and is managed under a single treatment algorithm in the advanced setting [3]. While G/GOJ adenocarcinoma makes up 95% of gastric cancer, oesophageal adenocarcinoma is the minority histological subtype of oesophageal cancer globally. Surgery and adjuvant or neoadjuvant systemic therapy remain the mainstay of treatment for early-stage or locally advanced GEA. For those diagnosed with advanced GEA, disease that is unresectable, recurrent or metastatic, median overall survival (mOS) is 13–20 months, making therapeutic developments increasingly important [4].

Targeted therapies in advanced GEA have emerged as a leading area of research since the ToGA trial demonstrated a statistically significant improvement in OS for patients with human epidermal growth factor receptor 2 (HER2)-positive advanced G/GOJ adenocarcinoma treated with the monoclonal antibody (mAb) trastuzumab [5]. Since this landmark trial, only a small number of non-immunotherapy targeted agents have been approved for use in advanced GEA. When employed alongside traditional chemotherapy and novel immunotherapy agents, these therapies have shown significant improvements in OS and progression-free survival (PFS) in the first-, second- and third-line settings [5,6,7]. Targeted therapies rely on biomarker testing, with clinical trials frequently failing due to poor selection of patients. In Europe, HER2, programmed death-ligand 1 (PD-L1) and claudin 18.2 (CLDN18.2) biomarkers are routinely screened for in advanced GEA. Microsatellite instability-high (MSI-H)/mismatch repair deficiency (dMMR) status is also assessed to tailor anticancer therapy. Emerging biomarkers FGFR2b and MET are being investigated. Challenges in developing novel therapies include tumour heterogeneity, which creates primary and acquired resistance to targeted treatment, and poor standardisation of biomarkers leading to challenges in interpretation and efficacy of clinical trials [8,9].

**Figure 1:**
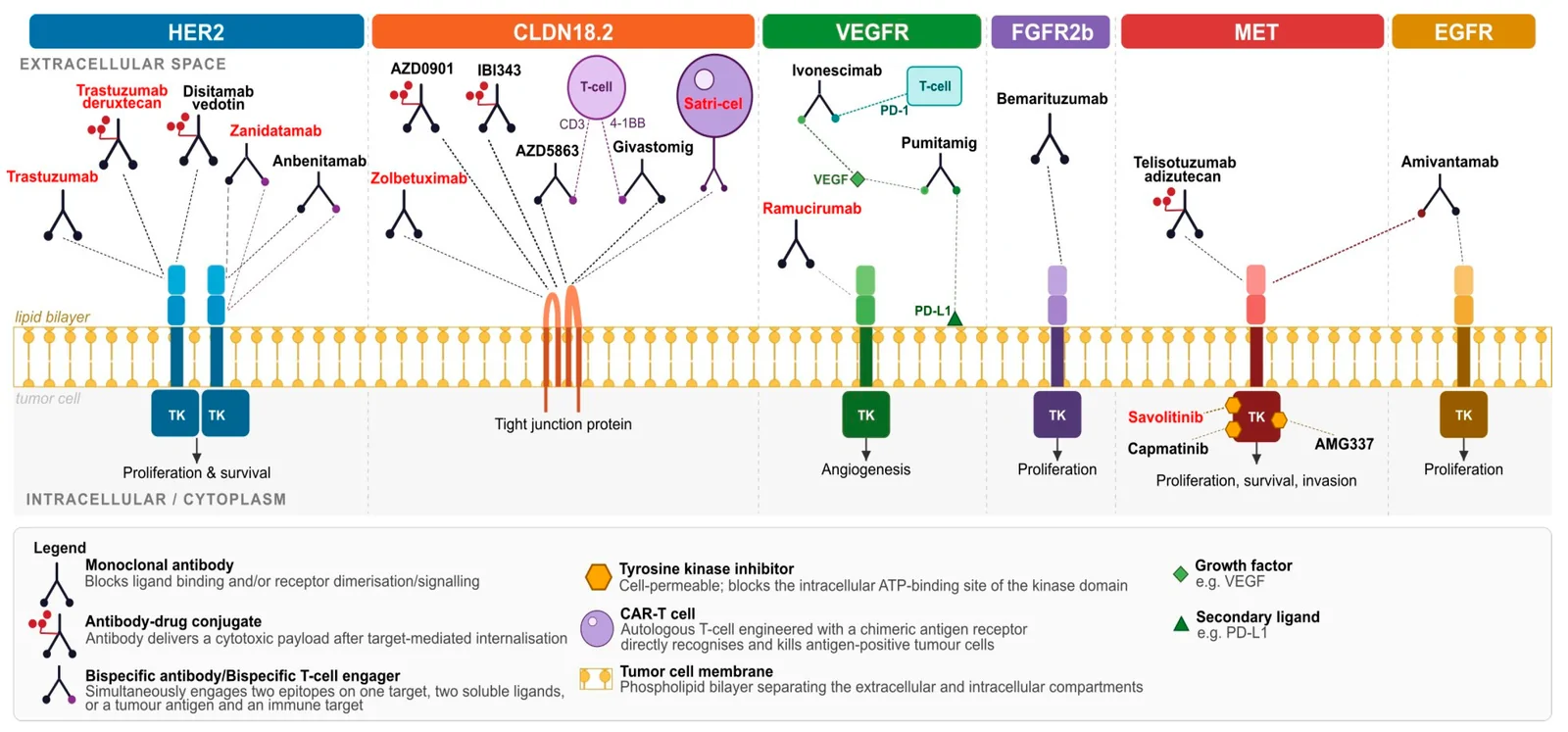
Schematic diagram of selected therapeutic targets and their corresponding target agent. Red font indicates approved treatments (does not indicate where approved, for more details, refer to Table 1).

This review comprehensively summarises current and emerging targeted therapies in the treatment of advanced GEA, including existing and novel techniques such as monoclonal antibodies (mAbs), bispecific antibodies, antibody–drug conjugates (ADCs), tyrosine kinase inhibitors (TKIs), bispecific T-cell engagers (BiTEs) and chimeric antigen receptor T-cell (CAR-T) therapy (Figure 1). Key trials and toxicities are summarised in Table 1. The review will highlight where studies primarily examine G/GOJ adenocarcinoma and use GEA where oesophageal adenocarcinoma is incorporated.

## 2. Methodology

A comprehensive review of validated sources not limited to PubMed, Google Scholar and Cochrane Library was conducted in line with PRISMA (preferred reporting items for systematic reviews and meta-analyses) guidelines. Peer-reviewed original research, systemic reviews, meta-analyses, abstracts, webpages and central guidelines were selected according to their relevance to the title subject and contribution to clinical practice. Only human studies published in English were reviewed. The databases were searched between 2 February and 30 July 2026, with some details updated during the editing process to remain in line with the latest standards.

Key search terms included “advanced gastroesophageal adenocarcinoma”, “gastroesophageal junction”, oesophageal adenocarcinoma”, “precision medicine”, “targeted therapy”, “HER2”, “CLDN18.2”, “VEGFR”, “FGFR2b”, “MET”, “EGFR”, “perioperative targeted therapy”.

## 3. Human Epidermal Growth Factor Receptor 2 (HER2)

HER2 belongs to the ErbB family of transmembrane glycoproteins with intrinsic tyrosine kinase activity, which includes HER1 (EGFR), HER3 and HER4. HER2 lacks a known endogenous ligand and instead functions as a preferred co-receptor for other family members. Upon activation, HER2 forms homo- or heterodimers with ErbB receptors, triggering downstream intracellular signalling cascades, including the PI3K/AKT/mTOR and RAS/MAPK pathways, which promote tumour cell proliferation, survival, adhesion, migration and differentiation.

HER2 overexpression occurs in approximately 15% of patients with advanced GEA, making it one of the most clinically actionable molecular targets in this disease [4]. Accurate HER2 testing is therefore necessary before initiating first-line therapy. Standard assessment follows a dual-modality algorithm: immunohistochemistry (IHC) scoring of 3+ is considered definitively positive, IHC 0 or 1+ is negative, whilst IHC 2+ requires confirmatory in situ hybridisation (ISH) to determine HER2 gene amplification status [35]. However, resistance mechanisms to HER2-targeted therapy in GEA ensure challenging management. These include phenotypic and genomic biomarker heterogeneity and downregulation, where HER2 loss occurs in 34.8–68.8% of initially HER2-positive G/GOJ adenocarcinomas [36].

### 3.1. Monoclonal Antibodies (mAbs)

Trastuzumab, a humanised IgG1 mAb targeting extracellular domain IV of HER2, has been used in combination with chemotherapy in the first-line for HER2-positive advanced G/GOJ adenocarcinoma since the ToGA trial demonstrated a significant increase in mOS from 11.1 to 13.8 months (hazard ratio [HR] 0.74, 95% confidence interval [CI] 0.60–0.91; *p* = 0.0046) and median PFS (mPFS) from 5.5 to 6.7 months. The most common adverse events in both groups were nausea, vomiting and neutropenia [5]. This landmark, phase 3, randomised controlled trial was the first to indicate the importance of targeted therapy in advanced GEA and set the benchmark for subsequent research.

Encouraged by the synergistic activity observed with dual HER2 blockade in breast cancer, combination therapy trastuzumab and pertuzumab was investigated in the phase 2 JACOB trial. Despite a numerically higher OS in the pertuzumab arm (17.5 vs. 14.2 months), the difference did not reach statistical significance (HR 0.84; *p* = 0.057), and pertuzumab has not been adopted in advanced GEA, which displays wider HER2 heterogeneity and less reliance on HER2 signalling than HER2-positive breast cancer [37].

More recent work has focused on the combination of trastuzumab with immune-checkpoint inhibitors (ICIs). The phase 3 KEYNOTE-811 trial investigated the addition of pembrolizumab (ICI) to trastuzumab and chemotherapy in the first-line treatment of advanced G/GOJ adenocarcinoma, demonstrating significantly higher ORR; 73% (95% CI, 68–78) in the pembrolizumab arm and 58% (95% CI, 53–64) in the placebo arm. Subsequent analysis also demonstrated improved PFS; 10.9 vs. 7.3 months (HR 0.72, 95% CI, 0.60–0.87), and OS, 20.0 vs. 16.8 months (HR 0.80, 95% CI, 0.67–0.94; *p* = 0.0040) in patients with PD-L1-positive tumours (combined positive score [CPS] ≥ 1) [10]. These findings prompted the Food and Drug Administration (FDA) and European Medicines Agency (EMA) to approve pembrolizumab + trastuzumab + fluoropyrimidine/platinum in the first-line HER2-positive, PD-L1 (CPS ≥ 1) setting for locally advanced or unresectable/metastatic G/GOJ adenocarcinoma [38,39].

### 3.2. Antibody–Drug Conjugates (ADCs)

ADCs are mAbs linked to cytotoxic payloads that are delivered specifically to cells expressing target receptors. Their design allows greater specificity in drug delivery, thereby maximising anti-tumour efficacy whilst limiting systemic toxicity. The GATSBY series was the first to utilise an ADC, trastuzumab emtansine (T-DM1), in G/GOJ adenocarcinoma, but failed to demonstrate an improved OS compared with chemotherapy [40]. This series again highlighted HER2 heterogeneity in GEA and demonstrated that additional kinases may compensate when HER2 alone is targeted due to chromosomal instability.

Trastuzumab deruxtecan (T-DXd) is a next-generation ADC comprising an anti-HER2 antibody conjugated to a topoisomerase I inhibitor. Following receptor-mediated internalisation, the linker is cleaved, releasing the cytotoxic payload. T-DXd has been observed to exert a potent ‘bystander effect’, where the payload can kill adjacent antigen-negative tumour cells [41]. T-DXd has been studied across the DESTINY-Gastric series [6,11,12,13]. DESTINY-Gastric01, a randomised controlled trial in Japan and South Korea, enrolled patients with HER2-positive advanced G/GOJ adenocarcinoma who were refractory to ≥2 lines of therapy, at least one of those being trastuzumab. T-DXd was compared against investigator-selected chemotherapy, and revealed an ORR of 51% vs. 14% and a significant OS advantage of 12.5 vs. 8.4 months (HR 0.59; *p* = 0.0097) [11]. The DESTINY-Gastric02 study was conducted in a similar patient population in the USA and Europe and demonstrated a significant objective response with a comparable safety profile [12]. DESTINY-Gastric04, a randomised phase 3 trial, has published findings that T-DXd significantly improves OS vs. ramucirumab and paclitaxel in the second-line setting (14.7 vs. 11.4 months, HR 0.70; *p* = 0.009) [13]. A key inclusion criterion for DESTINY-Gastric02 and 04 was continued HER2-positivity following trastuzumab-based first-line therapy as confirmed on a fresh biopsy. Following DESTINY-Gastric04, T-DXd was widely approved as a monotherapy for HER2-positive advanced G/GOJ adenocarcinoma after prior trastuzumab-based regimen [42]. Confirmation of continued HER2-positivity after first-line is recommended where feasible.

In the investigational first-line setting, DESTINY-Gastric03, a phase 1b/2 multi-centre, open-label, dose-escalation and expansion study, demonstrated a manageable safety profile in triplet (T-DXd, fluoropyrimidine and pembrolizumab) [6]. DESTINY-Gastric05 trial, a phase 3 trial assessing T-DXd in previously untreated patients, is recruiting and aims to challenge trastuzumab-based therapy in the first-line HER2-positive setting.

Disitamab vedotin (RC-48) combines a high-affinity anti-HER2 antibody with monomethyl auristatin E (MMAE) and has shown an immune-modulatory bystander effect, activating immune signalling through the cGAS-STING pathway [43]. A phase 1 trial has demonstrated the tolerability and preliminary efficacy in previously treated, advanced, HER2-positive G/GOJ adenocarcinoma [44].

Additional HER2-directed cytotoxic ADCs in development include IKS014 and MRG002. Immune-stimulating antibody conjugates such as BDC-1001 and SBT6050 represent a mechanistically distinct class.

### 3.3. Bispecific Antibodies

Novel bispecific antibodies are being developed for advanced GEA. Zanidatamab (ZW25) binds two specific HER2 epitopes (extracellular domains II and IV) causing clusters of HER2. In vitro, zanidatamab provokes potent complement-dependent cytotoxicity (CDC), HER2 downregulation and internalisation, antibody-dependent cellular cytotoxicity (ADCC) and antibody-dependent cellular phagocytosis (ADCP) [45]. A phase 2 trial enrolled patients in Canada, South Korea and the USA to investigate the combination of zanidatamab and chemotherapy in previously untreated HER2-positive advanced GEA and reported an ORR of 76.2% (95% CI, 60.5–87.9) with a median duration of response of 18.7 months (95% CI, 10.4–44.1), although interpretation is limited by the single-arm design. The most common grade 3–4 treatment-related adverse events (TRAEs) were diarrhoea and hypokalaemia [14].

The recently published HERIZON-GEA-01 phase 3 study combined zanidatamab with chemotherapy ± ICI vs. trastuzumab plus chemotherapy in the first-line for HER2-positive advanced GEA. Both zanidatamab-containing regimens improved mPFS to 12.4 months, compared with 8.1 months with trastuzumab plus chemotherapy. Zanidatamab plus tislelizumab and chemotherapy also significantly improved mOS to 26.4 months (HR 0.72, 95% CI, 0.57–0.90; *p* = 0.0043) vs. 19.2 months with trastuzumab plus chemotherapy, whereas a mOS of 24.4 months with zanidatamab plus chemotherapy had not reached statistical significance at the interim analysis. Additionally, analysis of the PD-L1 tumour area positivity (TAP) < 1% subgroup demonstrated a mOS of 29.7 vs. 15.8 months for the triplet combination vs. control, respectively. However, this analysis remains exploratory and does not definitively demonstrate benefit regardless of PD-L1 status [15].

Based on this landmark trial, the FDA approved zanidatamab with tislelizumab and chemotherapy as first-line therapy in advanced GEA, surpassing trastuzumab-based therapy in the HER2-positive setting [46].

Furthermore, anbenitamab (KN026), a novel HER2-targeted bispecific antibody, has been studied in both the first- and second-line settings in advanced G/GOJ adenocarcinoma. By binding extracellular domains II and IV of HER2, KN026 causes receptor clustering and enhances ADCC and CDC effects. Phase 2 trials reported a tolerable adverse event profile with effects on both HER2-high and HER2-low (IHC 2+/ISH-negative) populations, and comparable results to standard-of-care approaches in the second-line setting [16]. Published interim analysis of phase 3 trial KC-WISE evaluating KN026 in previously treated, HER2-positive G/GOJ adenocarcinoma has demonstrated statistically significant improvements in both mPFS (7.1 months, 95% CI, 5.5–10.3 months vs. 2.7 months, 95% CI, 1.5–3.0 months, HR 0.25, 95% CI, 0.17–0.39; *p* < 0.0001) and mOS (19.6 months, 95% CI, 15.0 months–not evaluable vs. 11.5 months, 95% CI, 6.5–14.4 months, HR 0.29, 95% CI, 0.17–0.50; *p* < 0.0001) than chemotherapy alone [17]. Confirmation at final analysis is awaited.

## 4. Claudin 18.2 (CLDN18.2)

CLDN18.2 is a tight junction protein expressed in normal gastric mucosa cells and is retained in most GEA [47]. During malignant transformation, loss of gastric mucosa cell polarity may result in CLDN18.2 becoming more exposed and more accessible to therapeutic antibodies [47,48]. Approximately 35–45% of patients with gastric cancer are CLDN18.2-positive [49,50].

### 4.1. Monoclonal Antibodies (mAbs)

Zolbetuximab is a first-in-class IgG1 mAb that targets CLDN18.2 and mediates ADCC and CDC in CLDN18.2-positive tumour cells. SPOTLIGHT was the first global, phase 3 study to examine the efficacy and safety of the addition of zolbetuximab to standard mFOLFOX6 in advanced G/GOJ adenocarcinoma; it demonstrated prolonged survival with a CLDN18.2-targeted therapy [7]. All eligible patients were HER2-negative and CLDN18.2-positive, which was defined as ≥75% of tumour cells showing moderate-to-strong membranous CLDN18 staining. Patients in the investigational arm achieved a significant PFS advantage over the placebo and mFOLFOX6 group (10.61 months vs. 8.67 months, HR 0.75, 95% CI, 0.60–0.94; *p* = 0.0066). An OS benefit was also observed among patients in the investigational arm (18.23 months vs. 15.54 months, HR 0.75, 95% CI, 0.60–0.94; *p* = 0.0053). ORR was 48% in both the zolbetuximab (95% CI, 42–54) and placebo groups (95% CI, 42–54). The median duration of response (DOR) was 9.00 months (95% CI, 6.87–10.25) in the investigational arm vs. 8.05 months (95% CI, 6.47–10.81) in the placebo arm of the study. The type and rate of subsequent anticancer therapies were similar among the zolbetuximab (48%) group vs. the placebo (53%) group. Frequently occurring grade ≥ 3 TRAEs were mainly comprised of decreased neutrophil count, nausea and vomiting. TRAEs led to discontinuation of zolbetuximab in 14% of patients and discontinuation of placebo in 2% of subjects.

Similar to the SPOTLIGHT trial, the double-blind, phase 3 GLOW trial explored the combination of zolbetuximab with CAPOX in the same clinical population within a first-line treatment setting [18]. Patients in the investigational zolbetuximab and CAPOX arm achieved a significant PFS advantage over subjects in the placebo and CAPOX arm (8.21 months vs. 6.80 months, HR 0.69, 95% CI, 0.54–0.87; *p* = 0.0007). At the interim analysis, OS was significantly prolonged among patients in the zolbetuximab group vs. the placebo group (14.39 months vs. 12.16 months, HR 0.77, 95% CI, 0.62–0.97; *p* = 0.0118). Nevertheless, the GLOW and SPOTLIGHT trials both demonstrated a similar reduction in the risk of disease progression or death (~31% and ~25%, respectively) and a similar reduction in the risk of death (~25% in both studies) with the addition of zolbetuximab to chemotherapy when compared with placebo plus chemotherapy. The consistent HRs observed in both GLOW and SPOTLIGHT represent a clinically meaningful benefit for patients with G/GOJ adenocarcinoma. Interestingly, similar ORR (42.5% vs. 40.3%) and DOR (6.14 months vs. 6.08 months) were noted in the zolbetuximab and placebo treatment groups, respectively. This observation highlights the notion that improvements in survival outcomes may not always be driven by differences in response dynamics between treatment groups [51]. Similar grade ≥ 3 TRAEs were reported, and comparable rates of treatment discontinuation and death were recorded in the zolbetuximab and placebo arms.

Recent pre-clinical and translational investigations have provided a biological rationale to drive a triplet combinatorial treatment approach leveraging complementary CLDN18.2 and PD-1 tumour biology. For instance, zolbetuximab-mediated cytotoxicity can promote tumour antigen release and dendritic cell priming, whereas co-inhibition of the PD-1 pathway may amplify tumour-specific T-cell response, thereby potentially inducing innate and adaptive immune responses [52,53]. This therapeutic concept has been explored as part of cohort 4 in the phase 2 ILUSTRO trial assessing first-line zolbetuximab, mFOLFOX and nivolumab in patients with HER2-negative, advanced G/GOJ adenocarcinoma whose tumours had either high (≥75% of tumour cells) or intermediate (≥50% and <75% of tumour cells) IHC CLDN18 staining [54]. Analyses of the expansion phase (cohort 4B) demonstrated a mPFS of 14.8 months (95% CI, 8.3—not estimable). Patients with high CLDN18.2 expression achieved a PFS of 18.0 months (95% CI, 11.1—not estimable). Among patients with high CLDN18.2 expression and available PD-L1 results, 36 had PD-L1 CPS ≥ 1; mPFS in this subgroup was 23.6 months, compared with 12.1 months among the 21 patients with CPS < 1. OS data were immature at the time of analysis. With a median follow-up time of 15.4 months, the mOS was 18.0 months (95% CI, 13.6—not estimable) in all patients in cohort 4B and not estimable (95% CI, 13.7—not estimable) among subjects with high CLDN18.2 tumour expression. In patients with measurable disease at baseline, the ORR in cohort 4B of 58 patients was 62.1% (95% CI, 48.4–74.5), including 2 (3.4%) CRs; these responses were largely driven by the main CLDN18.2 high cohort of 47 (81%) patients.

Although these findings indicate encouraging preliminary antitumour activity with the combination, their interpretation is limited by the non-randomised phase 2 design, the relatively small subgroup sizes, and immature OS data. In particular, the observed differences according to PD-L1 expression should be considered hypothesis-generating rather than evidence of differential treatment efficacy. Accordingly, these data provide preliminary clinical support for further investigation of combined CLDN18.2 and PD-1 pathway inhibition but do not establish superior efficacy relative to existing treatment approaches. The potential clinical benefit of this strategy is being evaluated in the ongoing global, randomised, phase 3 LUCERNA trial evaluating zolbetuximab, CAPOX or mFOLFOX6 with pembrolizumab in patients with HER2-negative, advanced G/GOJ adenocarcinoma with CLDN18.2-positive and PD-L1 CPS ≥ 1 tumours (NCT06901531).

### 4.2. Antibody–Drug Conjugates (ADCs)

With the development of novel CLDN18.2-targeting ADCs, CLDN18.2 is now becoming an active target for drug delivery alongside an antibody target. The first of these ADCs is AZD0901 (CMG901 or sonesitatug vedotin), a humanised anti-CLDN18.2 antibody linked to MMAE. The phase 1, dose-escalation and expansion KYM901 trial demonstrated antitumour activity and a manageable safety profile [19]. It is being followed by CLARITY-Gastric01, a phase 3 randomised study of AZD0901 in second- or later-line advanced G/GOJ adenocarcinoma vs. investigator’s choice of therapy, which is in recruitment [20].

Moreover, IBI343, a fully humanised anti-CLDN18.2 mAb conjugated to exatecan, is in development. A phase 1 trial in advanced G/GOJ adenocarcinoma demonstrated an ORR of 29% (95% CI , 14.2–48.0) and mPFS of 5.5 months (95% CI, 4.1–7.0) in CLDN18.2-high disease (2+/3+ staining on ≥75% tumour cells), and a manageable safety profile with minimal gastrointestinal adverse events (grade ≥ 3). No responses were seen in the CLDN18.2-low group, reinforcing the importance of CLDN18.2 expression level [21].

Other CLDN18.2-targeting ADCs include SHR-A1904 and LM-302 (tecotabart vedotin). Collectively, these programmes broaden CLDN18.2 from an antibody target to a payload-delivery antigen, while also testing how expression level and intratumoural heterogeneity affect its durability as a therapeutic target.

### 4.3. Bispecific T-Cell Engagers (BiTEs)

Beyond ADCs, CLDN18.2 can similarly serve as a target for immune cell engagements. AZD5863 is a bispecific T-cell engager with a high-affinity binding domain for CLDN18.2 and a low-affinity domain for cluster of differentiation 3 (CD3), designed to promote T-cell-mediated tumour-cell killing while potentially minimising systemic immune activation. In vitro and in vivo studies have demonstrated lower cytokine release with AZD5863 than with a T-cell engager incorporating stronger CD3 binding, whilst bystander killing of CLDN18.2-negative tumour cells has also been observed in the presence of CLDN18.2-expressing cells [23]. These findings provide preclinical support for the mechanism of action; however, the clinical efficacy and safety of AZD5863 remain under investigation in an ongoing phase 1/2 dose-escalation and expansion study in adults with advanced or metastatic solid tumours (NCT06005493).

The bispecific antibody givastomig utilises a different immune-engaging response by conditionally activating local 4-1BB-expressing T-cells. 4-1BB is a co-stimulatory molecule with the ability to directly stimulate CD8^+^ T-cell proliferation and enhance cytotoxicity. Givastomig is designed to activate immune cells preferentially following engagement of CLDN18.2, with the aim of limiting systemic immune-related toxicity. Preliminary data from a phase 1b study combining givastomig, nivolumab and mFOLFOX in HER2-negative, CLDN18.2-positive advanced GEA demonstrated a 71% ORR. Grade ≥ 3 abdominal pain, liver function marker elevation, gastritis and infusion-related reaction were each reported in single cases [22]. These early findings indicate promising preliminary antitumour activity and provide clinical support for further investigation, but the efficacy and safety of givastomig relative to established treatment approaches remain to be determined in comparative studies.

### 4.4. Chimeric Antigen Receptor (CAR) T-Cells

Finally, CLDN18.2 has become a target for CAR-T-cell therapy, which uses autologous, genetically modified T-cells to recognise and attack target cancer cells. While CAR-T therapy is widely used in haematological malignancies, there has been limited success in vivo in the solid tumour setting.

Satricabtagene autoleucel (satri-cel) is the first CAR-T therapy worldwide to enter randomised controlled trials in solid tumours. A phase 1 dose-escalation and expansion trial studied satri-cel in previously treated gastrointestinal cancers with at least one line of standard systemic therapy. Key grade ≥ 3 TRAEs were haematological toxicities, which typically recovered within 6–14 days. Cytokine release syndrome (CRS) grade 1 or 2 occurred in 96.9% of patients, with no grade 3 or higher CRS observed. No immune effector cell-associated neurotoxicity syndrome (ICANS), haemophagocytic lymphohistiocytosis (HLH) or treatment-related death was observed. Finally, expected gastric mucosal injuries occurred in 8.2% of patients [55]. Together, these represent a manageable safety profile.

Following this, a phase 2 randomised controlled trial was developed to compare satri-cel with physician’s choice therapy in previously treated advanced G/GOJ adenocarcinoma. Satri-cel demonstrated a statistically significant improvement in mPFS to 3.25 months from 1.77 months in the physician’s choice group (HR 0.37, 95% CI, 0.24–0.56; one-sided log-rank *p* < 0.0001). However, the improved mOS from 5.49 to 7.92 months had a 95% CI that included 1.0 (HR 0.69, 95% CI 0.46–1.05; one-sided log-rank *p* = 0.0416). Additionally, grade ≥ 3 TRAEs were seen in 99% of patients in the satri-cel group, mostly haematological toxicities, with four cases of CRS graded ≥ 3 and one death due to disseminated intravascular coagulation [24]. The final analysis of mOS alongside a significant toxicity profile requires careful interpretation of these phase 2 results.

Nevertheless, satri-cel received National Medical Products Administration (NMPA) approval in China in June 2026 for CLDN18.2-positive, HER2-negative advanced G/GOJ adenocarcinoma after failure of at least two previous lines [56]. It is the first approved CAR-T-cell product for a solid tumour.

## 5. Vascular Endothelial Growth Factor Receptor 2 (VEGFR2)

Vascular endothelial growth factor (VEGF) has a key role to play in tumour regulation of angiogenesis and vascular permeability, which assist in tumour growth, invasion and metastatic spread in GEA. VEGF-A-VEGFR-2 signalling is relevant, as VEGFR2 is the main driver in pro-angiogenic receptor tyrosine kinase-mediated endothelial proliferation and the formation of new blood vessels. The main mechanism of action has aimed at targeting ligand neutralisation (anti-VEGF) or blockade of the receptor (anti-VEGFR2). This has demonstrated a notable impact in the treatment of GEA, especially via mAbs such as VEGFR-2 inhibitors, which abrogate downstream signalling cascades comprising PLCγ–PKC–MAPK/ERK, Src–PI3K–AKT and p38/FAK pathways [25,57,58].

### 5.1. Monoclonal Antibodies (mAbs)

Ramucirumab is a mAb that binds to the extracellular component of VEGFR2, thereby preventing VEGF ligand-receptor activation and angiogenic signalling [25,26,58]. It has been evaluated extensively and has established itself as a cornerstone anti-angiogenic therapy in this disease setting.

In the phase 3 REGARD trial, ramucirumab increased mPFS from 1.3 months to 2.1 months compared with the placebo group (HR 0.48, 95% CI, 0.38–0.62; *p* < 0.0001). A modest improvement in OS was observed from 3.8 months in the placebo group to 5.2 months in the investigational arm (HR 0.78, 95% CI, 0.60–1.00; *p* = 0.047). ORRs remained low and were similar between groups (3% vs. 3%), whereas the disease control rate (DCR) was higher and statistically significant, with ramucirumab use (49% vs. 23%; *p* < 0.0001). Subgroup analyses suggested generally consistent PFS and OS benefit across most patient groups, although exploratory biomarker analyses did not identify a clear predictive biomarker of response [25]. Inhibition of the VEGF axis was linked to expected but generally manageable toxicity. Grade ≥ 3 hypertension was more prevalent with ramucirumab, followed by increased abdominal pain. Treatment-related deaths occurred in 2% of patients in both the ramucirumab and placebo cohorts.

In the phase 3 RAINBOW trial, the combination of ramucirumab and paclitaxel improved outcomes in patients with advanced G/GOJ adenocarcinoma who were progressing after first-line platinum/fluoropyrimidine therapy. The trial demonstrated superior OS (9.6 months vs. 7.4 months, HR 0.81; *p* = 0.017) and a significantly elevated DCR in the investigational arm compared to the placebo plus paclitaxel cohort (80% vs. 64%; *p* < 0.0001) [26]. However, the treatment combining ramucirumab with paclitaxel demonstrated an increase in grade ≥ 3 neutropenia (41% vs. 19%), leukopenia (17% vs. 7%) and hypertension (14% vs. 2%).

Bevacizumab targets VEGF at the ligand level but did not demonstrate a significant clinical benefit in the G/GOJ adenocarcinoma patient population. This demonstrated that the efficacy of angiogenic pathway inhibition may be more contingent upon other factors such as treatment line, chemotherapy partner, and the inherent tumour biology [57].

Ramucirumab received FDA approval for previously treated advanced/metastatic gastric or G/GOJ adenocarcinoma based on the RAINBOW and REGARD studies [58].

### 5.2. Bispecific Antibodies

Novel bispecific antibodies are in development targeting both VEGF and PD-1/PD-L1 and aim to block immune escape and VEGF-mediated immune suppression simultaneously.

Clinical rationale of this approach has so far come principally from the phase 3 HARMONi-6 trial investigating ivonescimab, a first-in-class bispecific antibody targeting PD-1 and VEGF, in advanced squamous non-small cell lung cancer (NSCLC) [59]. However, it remains investigational in GEA and is being evaluated in dedicated trials such as UCGI52–Gracie [60].

Moreover, pumitamig (BNT327/PM8002) is an investigational bispecific antibody targeting VEGF and PD-L1 simultaneously that has shown antitumour efficacy in early-phase trials. The phase 2/3, global, blinded ROSETTA Gastric-204 trial is investigating pumitamig with chemotherapy against the standard-of-care nivolumab with chemotherapy in the first-line for patients with advanced GEA and PD-L1 ≥ 1 (NCT07221149).

Although investigational, these treatments are demonstrating antitumour activity with manageable safety profiles and include patients who had been biomarker-negative for immunotherapy.

## 6. Fibroblast Growth Factor Receptor (FGFR2b)

Patients overexpressing FGFR2b represent a biomarker-defined subset of advanced GEA characterised by aggressive biology and potential oncogenic dependency [27,61]. In GEA, FGFR2b (FGFR2-IIIb) is an epithelial receptor that is often activated by fibroblast growth factor 7 (FGF7) from stromal fibroblasts. This results in receptor dimerization and auto-phosphorylation, which initiates the PI3K–Akt–mTOR signalling cascade. Such tumours exhibit elevated FGFR2 expression compared to the adjacent mucosa, which correlates with deeper invasion and a more advanced disease stage. Functionally, FGF7–FGFR2 signalling facilitates migration and invasion, partially through the induction of thrombospondin-1 (THBS1) secretion. Inhibition of FGFR2 or THBS1 diminishes these invasive phenotypes [62].

### Monoclonal Antibodies (mAbs)

Bemarituzumab (FPA144) is a humanised IgG1 anti-FGFR2b mAb that has been afucosylated. It abrogates FGFR2b signalling and is designed to improve immune effector function by increasing Fc–Fcγ receptor interactions [27,63]. In the randomised, phase 2 FIGHT trial for first-line FGFR2b-selected G/GOJ adenocarcinoma, the combination of bemarituzumab and mFOLFOX6 led to extension of mOS to 19.2 months compared to 13.5 months in the placebo plus mFOLFOX6 group (HR 0.77, 95% CI, 0.52–1.14) with a more pronounced clinical benefit in tumours with an elevated FGFR2b expression (≥10% FGFR2b-positive cells), supporting this expression threshold as a potential criterion for future patient selection [27]. Studies identified that bemarituzumab can cause eye and corneal adverse events, often prompting a dose change or cessation of treatment.

Following FIGHT, the phase 3 FORTITUDE-101 trial investigated bemarituzumab and mFOLFOX6 vs. placebo and mFOLFOX6 in first-line FGFR2b-overexpressing (≥10%) G/GOJ adenocarcinoma. Unlike FIGHT, final analysis showed converging mOS of 14.5 vs. 13.2 months (HR 0.82), despite improved mOS at interim analysis, therefore showing a weakened signal with longer follow-up [28]. FORTITUDE-102 explored whether adding nivolumab would elucidate more sustainable improvements in mOS but was stopped for inadequate efficacy [29]. The unique eye and corneal adverse events raised the threshold for efficacy to justify the side-effect profile. FGFR2b remains an investigational biomarker in GEA, and following FORTITUDE-101 and subsequent termination of FORTITUDE-102, its clinical relevance remains uncertain.

## 7. MET

MET (also known as c-MET or hepatocyte growth factor receptor, HGFR) is a receptor tyrosine kinase encoded by the *MET* proto-oncogene, which has been implicated in tumour biology including proliferation, survival, epithelial-to-mesenchymal transition, angiogenesis and metastasis. It binds to its ligand, HGF, which is secreted into the tumour microenvironment to cause phosphorylation and activation of downstream signalling pathways [64]. MET dysregulation can occur through gene amplification, activating mutations, transcriptional upregulation, overexpression and autocrine/paracrine signalling loops [65]. MET has primarily been studied in the context of NSCLC where MET-selective TKIs have been licensed. *MET* proto-oncogene amplification is an oncogenic driver of 4–7% GEA and has been associated with worse OS and PFS [31].

MET targeting is complicated by wide heterogeneity, resistance and lack of standardisation in identification. These biological complexities are illustrated by the VIKTORY trial, which enrolled 715 patients into their biomarker screening programme. MET IHC was evaluable in 479, of which 42 had IHC 3+ expression. A total of 17 of these 42 also had *MET* amplification as seen by next-generation sequencing (NGS), and 25 did not [30]. This demonstrated that MET can be co-activated and does not require gene amplification to be overexpressed, thus underlining the challenges in patient selection.

### 7.1. Tyrosine Kinase Inhibitors (TKIs)

MET-selective TKIs have had more success than mAbs, and accordingly savolitinib received conditional NMPA approval in China in July 2026 for *MET*-amplified advanced G/GOJ adenocarcinoma following previous systemic therapy [66].

The previously mentioned phase 2 VIKTORY umbrella trial sequenced patients into molecular-driven subgroups, allowing biomarker-targeted second-line therapies in advanced gastric cancer. It reported an ORR of 50% (95% CI, 28.0–71.9) in patients receiving savolitinib monotherapy, substantially higher than existing benchmarks. One patient achieved complete response and underwent curative resection, achieving pathologic downstaging from M1 disease to T3N2M0 [30].

Furthermore, a pivotal phase 2 trial in China selected patients with *MET*-amplification (gene copy number (GCN) ≥ 10) advanced G/GOJ adenocarcinoma that had progressed following ≥2 prior lines of treatment to receive savolitinib orally. Of the 65 patients enrolled in the pivotal phase, an ORR of 32.3% (95% CI, 21.2–45.1%) was recorded, which reached its primary endpoint (ORR ≥ 15%). The study used FISH to determine GCN and, alongside VIKTORY, confirms the role of strict patient selection for savolitinib therapy. The study was limited by its single-arm and non-randomised design but has proven a durable antitumour response in pre-treated *MET*-amplification G/GOJ adenocarcinoma for prospective randomised controlled trials [31].

Other MET-TKIs include capmatinib and AMG337, the former having been licensed for use in NSCLC. In phase 1 trials, capmatinib achieved stable disease in two out of nine patients with gastric cancer [67] and AMG337 confirmed 29.7% (95% CI, 13.8–50.2%) ORR in *MET*-amplified GEA [68]. In phase 2 trials, AMG337 yielded 18% ORR (95% CI, 8–32%) in 45 patients with *MET*-amplified GEA [69].

### 7.2. Antibody–Drug Conjugates (ADCs)

Telisotuzumab adizutecan (Temab-A or ABBV-400) combines an anti-MET mAb with a topoisomerase I payload and has shown activity in a variety of solid tumours in phase 1 trials through mechanisms analogous to T-DXd. In patients with advanced GEA, an ORR of 28.6% was observed. TRAEs grade ≥ 3 occurred in 88.1% of patients, the most common being anaemia [32]. A combination of Temab-A with 5-FU, leucovorin and a PD-1 blocking antibody is now being studied in the first-line for HER2-negative advanced GEA (NCT06628310).

## 8. Epidermal Growth Factor Receptor (EGFR)

Epidermal growth factor receptor (EGFR or ERBB1) is another member of the ErbB family and is overexpressed in approximately 30% of GEA [70]. EGFR binds multiple ligands, including epidermal growth factor (EGF), transforming growth factor-α (TGF-α), amphiregulin, and epiregulin, to induce survival and proliferation signalling through the MAPK, STAT5 and Ras/Raf/MEK pathways [71]. Out of the overexpressed proteins, increased EGFR are most associated with a higher stage, more poorly differentiated tumour with increased vascular invasion [72].

Despite common overexpression in GEA, trials into mAb and TKI therapies such as cetuximab (EXPAND), panitumumab (REAL3) and gefitinib (COG/TRANS-COG) have not shown meaningful clinical benefit [73,74,75]. This has been attributed to the unselected populations and absence of validated predictive biomarkers.

### Bispecific Antibodies

Amivantamab is a fully human bispecific antibody targeting both EGFR and MET, incorporating an Fc region engineered to enhance immune cell-directing activity, including ADCC and ADCP [76]. A phase 2 study of amivantamab in patients with previously treated, advanced GEA revealed an ORR of 4.3%, which increased to 13.3% in the cohort with higher MET expression (IHC 2+ or greater) [33]. Following this, AMIGO, a phase 2 trial of amivantamab monotherapy in the second-line for EGFR or *MET*-amplified GEA has recently reported a DCR of 71% with mPFS and mOS at 3.3 (95% CI, 2.1–7.7) and 8.8 months (95% CI, 5.7–not reported), respectively [34]. These results confirm the need for rigorous population selection. Monotherapy in GEA is consistently hampered by heterogeneity of targets leading to rapid resistance and expansion of cells with untargeted antigen expression such as FGFR or VEGFR. Future work will combine amivantamab with chemotherapy, anti-angiogenic agents or ICIs and several trials are in development.

## 9. Discussion

Targeted precision therapies have an increasingly important role in the treatment of advanced GEA. Only a small number of non-immunotherapy targeted agents have been approved globally, but the advent of new ADCs, bispecific antibodies, BiTEs and novel combinations with ICIs represent new pathways for future research.

In the context of HER2-mediated disease, zanidatamab could now become first-line standard-of-care following recent FDA approval. T-DXd is established second-line standard-of-care as a single agent, and the outcome of DESTINY-Gastric05 is anticipated, exploring T-DXd in the first-line. In this rapidly moving arena, the approval of additional first-line therapies will allow greater physician choice and enhance shared decision-making. The clinical success of HER2-directed ADCs has further expanded the therapeutic landscape, with multiple next-generation bispecific antibodies and ADCs entering clinical development. HER2 remains an ongoing research avenue; however, there remain significant challenges, including heterogeneity in GEA and downregulation following first-line therapy.

While the focus of research into targeted agents has been mostly on advanced GEA, utilising HER2-targeting therapies in the perioperative setting has attracted attention. The NEOHX and HER-FLOT trials demonstrated improved outcomes when combining trastuzumab with neoadjuvant chemotherapy in a HER2-positive patient population. In small patient cohorts, NEOHX reported a statistically significant 18-month disease-free survival of 71%, while HER-FLOT achieved R0 resection in 92.9% of patients [77,78]. More recent work has studied the dual HER2 blockade of trastuzumab and pertuzumab in the neoadjuvant setting with varying success. The phase 2 EORTC INNOVATION randomised patients into three perioperative cohorts: chemotherapy, chemotherapy plus trastuzumab and chemotherapy plus trastuzumab plus pertuzumab. Non-significant advantages in OS and PFS were seen in the trastuzumab plus chemotherapy group before the chemotherapy protocol was amended mid-study, but these were lost after the amendment [79]. Despite these results, harnessing targeted therapies in the perioperative setting remains an active area of development, with strategies focusing on newer-generation agents such as T-DXd and anti-CLDN18.2 agents.

CLDN18.2 has become a heavily investigated target with the development of BiTE and CAR-T-directed therapy. These mechanisms have shown proof of principle of the efficacy of CAR-T therapy in solid tumours, and satri-cel is the first solid tumour CAR-T therapy to be approved globally. However, its significant safety profile associated with immune activation alongside a non-significant OS improvement may slow approval in the United States (US) and Europe. Early steps away from zolbetuximab towards ADCs prove CLDN18.2 is becoming a delivery antigen as well as an antibody target, although these agents remain investigational.

MET and EGFR are promising targets for novel therapies. The development of MET and EGFR-specific drugs has proven successful in certain cancer types but highlights the importance of good patient selection in GEA due to tumour heterogeneity, which allows activation of non-targeted kinases. The lack of international standardisation in tumour marker selection will continue to stunt improvements in these fields, and the complexity of this process using IHC, NGS and FISH was highlighted by *MET* amplification vs. MET tyrosine kinase expression discrepancy in the VIKTORY trial. Bemarituzumab was a biologically interesting molecule targeting FGFR2b, but its relevance remains uncertain based on the findings of FORTITUDE-101 and 102.

Other notable targets such as TROP2 and CAPRIN1 are being investigated in the advanced GEA setting. TROP2-directed ADCs such as sacituzumab tirumotecan have been studied in phase 2 trials. CAPRIN1 is a cytoplasmic RNA-binding protein that is not expressed on the cell surface of non-transformed cells but is strongly and reproducibly expressed on the cell surface of many cancer types, including gastric, breast, lung, colon and renal carcinomas [80]. TRK950, a CAPRIN1-targeting mAb, is being studied in a phase 2, CAPRIN1-positive, advanced GEA setting (NCT06038578). Further targets are in development.

Future studies will also focus on the development of cancer vaccines that aim to overcome immune suppression imposed by malignancy and the tumour microenvironment [81]. Liquid biopsies and circulating tumour DNA (ctDNA) could have multiple uses in diagnosis, therapeutic monitoring and prognosis of different cancers. As small fragments of DNA that are released in tumour apoptosis, necrosis or active release, ctDNA harbours representative mutations for biomarker analysis and can indicate the presence of tumour cells after curative surgery or radiotherapy, replacing the often-non-specific tumour markers currently in use [82]. The DECIPHER phase II trial is designed to investigate whether early treatment with T-DXd in ctDNA-positive patients following surgery or chemotherapy will reduce micrometastatic disease. Additionally, ctDNA could provide a non-invasive alternative to re-biopsy following initial targeted therapy, such as determining HER2 status following trastuzumab to decide eligibility for T-DXd [83].

There will be ongoing challenges, notably heterogeneity and classification of gastric cancers, standardisation of the rapidly increasing tumour markers, resistance to therapy and adverse drug reactions. Moreover, wide adverse event profiles require a high index of suspicion and early involvement of the multidisciplinary team. T-DXd is noted for its interstitial lung disease or pneumonitis; bemarituzumab for ocular toxicity; VEGF-axis agents for hypertension and proteinuria; and zolbetuximab for gastrointestinal toxicity. While guidelines now exist for immunotherapy-related toxicities, there remains a lack of consensus for managing nuanced precision medicine adverse events, hampering patient recovery and treatment.

In conclusion, in this rapidly evolving landscape, developments in HER2 and CLDN18.2 are treatment-changing through zanidatamab, T-DXd and zolbetuximab. Further drug mechanisms remain investigational, such as ADCs targeting CLDN18.2 and bispecific antibodies dual-targeting VEGF and PD-1/PD-L1. In Europe and the US, both EGFR and MET remain exploratory, along with CLDN18.2-directed BiTEs and CAR-T therapy. The NMPA approval of satri-cel in advanced GEA represents a global first for CAR-T cell therapy in solid tumours and further development in this area is anticipated.

## Figures and Tables

**Table 1:** Selected clinically relevant established and emerging targeted therapies in advanced gastroesophageal adenocarcinoma.

Target	Agent and Class	Mechanism of Action	Key Trial(s)	Characteristic Toxicities	Approval Status
**HER2**	**Trastuzumab** mAb	Binds HER2 extracellular domain IV, inhibits receptor signalling and mediates ADCC.	ToGA, **phase 3**, first line: improved overall survival with chemotherapy [5]. KEYNOTE-811: pembrolizumab + trastuzumab + chemotherapy improved PFS and OS in PD-L1 CPS ≥1 disease [10].	Infusion reactions; asymptomatic left ventricular ejection fraction (LVEF) fall/cardiomyopathy; rare pneumonitis. Toxicities from chemotherapy or immunotherapy partners also apply.	**Approved globally** with pembrolizumab and chemotherapy for first-line, HER2-positive, PD-L1 (CPS ≥ 1) advanced G/GOJ adenocarcinoma.
**HER2**	**Trastuzumab deruxtecan** (**T-DXd**) ADC	HER2 antibody linked through a cleavable linker to a topoisomerase I inhibitor; high drug-to-antibody ratio and membrane-permeable payload produce a bystander effect.	DESTINY-Gastric01/02 established activity after trastuzumab [11,12]. DESTINY-Gastric04, **phase 3**: OS 14.7 vs. 11.4 months versus ramucirumab–paclitaxel [13].	Nausea, fatigue, anaemia, neutropenia and thrombocytopenia; interstitial lung disease/pneumonitis; rare LVEF decline.	**Approved globally** as monotherapy in second-line HER2-positive advanced G/GOJ adenocarcinoma after prior trastuzumab.
**HER2**	**Zanidatamab** Bispecific antibody	Binds HER2 extracellular domains II and IV, causing receptor clustering, internalisation and downregulation, with ADCC, ADCP and CDC.	**Phase 2** activity with chemotherapy [14]. HERIZON-GEA-01, **phase 3**: improved PFS with zanidatamab + chemotherapy with or without tislelizumab; OS benefit with the tislelizumab triplet [15].	Diarrhoea, nausea/vomiting, hypokalaemia, infusion-related reactions and anaemia; cardiac monitoring remains appropriate.	**Approved by the FDA** with tislelizumab and chemotherapy in first-line HER2-positive advanced GEA.
**HER2**	**Anbenitamab** (**KN026**) Bispecific antibody	Dual-epitope HER2 binding to domains II and IV, promoting receptor clustering and immune-mediated killing through ADCC and complement activation.	**Phase 2** studies showed activity in HER2-high and selected HER2-low disease [16]. KC-WISE, **phase 3**, reported improved PFS and OS versus chemotherapy in previously treated HER2-positive GEA [17]; final full publication is awaited.	Diarrhoea, infusion reactions, nausea/vomiting and cytopenias; possible HER2-class cardiac dysfunction.	Investigational.
**CLDN18.2**	**Zolbetuximab** mAb	Binds tumour-exposed CLDN18.2 and induces ADCC and CDC.	SPOTLIGHT and GLOW, **phase 3**, firstline: improved PFS and OS with mFOLFOX6 or CAPOX [7,18].	Prominent nausea and vomiting, reduced appetite and infusion reactions, particularly during the first treatment cycles.	**Approved globally** with chemotherapy for first-line CLDN18.2-positive, HER2-negative advanced G/GOJ adenocarcinoma.
**CLDN18.2**	**AZD0901** (**CMG901; sonesitatug vedotin**) ADC	CLDN18.2 antibody linked to MMAE; internalisation releases a microtubule-disrupting payload.	KYM901, **phase 1**: confirmed response rate approximately 29% in advanced G/GEJ cancer [19]. CLARITY-Gastric01, **phase 3**, is evaluating second- or later-line treatment [20].	Vomiting, reduced appetite, proteinuria, anaemia and neutropenia; MMAE-class peripheral neuropathy; pancreatitis was rare.	Investigational.
**CLDN18.2**	**IBI343** ADC	CLDN18.2 antibody conjugated to an exatecan-derived topoisomerase I inhibitor, permitting intracellular payload release and bystander killing.	**Phase 1**: response rate 29% in CLDN18.2-high GEA, with no responses in the low-expression cohort [21].	Anaemia, neutropaenia, nausea/vomiting and fatigue; interstitial lung disease is a potential topoisomerase I ADC class risk.	Investigational.
**CLDN18.2**	**Givastomig** BiTE	Givastomig conditionally co-stimulates 4–1BB-positive T-cells after CLDN18.2 binding.	Early phase development. Givastomig + nivolumab + mFOLFOX produced a high preliminary response rate in **phase 1b** [22].	Cytokine release/infusion reactions, fever and transaminitis; gastritis or abdominal pain. Frequency and severity depend on the construct and CD3/4-1BB activity.	Investigational.
**CLDN18.2**	**AZD5863** BiTE	AZD5863 links CLDN18.2-positive cells to CD3-positive Tcells to trigger cytotoxicity.	AZD5863 is in **phase 1/2** dose escalation and expansion [23].	Cytokine release syndrome, gastritis and abdominal pain.	Investigational.
**CLDN18.2**	**Satricabtagene autoleucel (satri-cel)** Autologous CAR-T-cell	Autologous T cells genetically engineered to recognise CLDN18.2 and kill antigen-expressing tumour cells.	Randomised **phase 2**: PFS 3.25 vs. 1.77 months and OS 7.92 vs. 5.49 months versus physician’s choice [24].	Lymphodepletion-related cytopaenias, cytokine release syndrome; gastric mucosal injury; rare severe coagulopathy. ICANS has been uncommon.	**Approved by the NMPA** for CLDN18.2-positive, HER2-negative advanced G/GOJ adenocarcinoma after ≥2 treatment lines.
**VEGFR2**	**Ramucirumab** mAb	Binds VEGFR2 and prevents VEGF ligand activation, suppressing tumour angiogenesis.	REGARD, **phase 3**, showed benefit as monotherapy [25]. RAINBOW, **phase 3**, established ramucirumab + paclitaxel as a second-line standard [26].	Hypertension, proteinuria, bleeding or thrombosis, impaired wound healing and gastrointestinal perforation; neutropaenia is increased with paclitaxel.	**Approved globally** as monotherapy or with paclitaxel for second-line advanced G/GOJ adenocarcinoma.
**VEGF + PD-(L)1**	**Pumitamig** Bispecific antibody	Simultaneous checkpoint blockade and VEGF neutralisation, aiming to inhibit angiogenesis and reverse VEGF-mediated immune suppression within the tumour.	ROSETTA Gastric-204 is comparing pumitamig + chemotherapy with nivolumab + chemotherapy in **phase 2/3** (NCT07221149).	Immune-related adverse events together with hypertension, proteinuria, bleeding/thrombosis and infusion reactions.	Investigational.
**FGFR2b**	**Bemarituzumab** mAb	Blocks FGFR2b signalling and enhances Fc-mediated ADCC through afucosylation.	FIGHT, **phase 2**, showed the strongest signal with ≥10% FGFR2b-positive tumour cells [27]. FORTITUDE-101 met its **phase 3** OS endpoint, but the magnitude attenuated with follow-up; FORTITUDE-102 was stopped for inadequate efficacy [28,29].	Corneal epithelial toxicity/keratitis, dry eye and blurred vision; stomatitis and infusion reactions. Early ophthalmology involvement is important.	Investigational.
**MET**	**Savolitinib** TKI	ATP-competitive inhibition of MET kinase signalling in MET-amplified tumours.	VIKTORY and a Chinese pivotal **phase 2** study reported response rates of approximately 32–50% in stringently selected MET-amplified GEA [30,31].	Nausea/vomiting, peripheral oedema, fatigue, transaminitis and cytopaenias; interstitial lung disease is rare.	**Approved by the NMPA** for MET-amplified advanced G/GOJ adenocarcinoma after ≥2 previous systemic treatments.
**MET**	**Telisotuzumab adizutecan (ABBV-400; Temab-A)** ADC	MET-directed antibody linked to a topoisomerase I inhibitor; internalisation releases a membrane-permeable payload with bystander activity.	**Phase 1** across solid tumours showed a response rate of approximately 29% in advanced GEA [32]. Monotherapy and combination studies are ongoing.	Anaemia, neutropaenia, nausea and fatigue; interstitial lung disease/pneumonitis requires surveillance.	Investigational.
**EGFR/MET**	**Amivantamab** Bispecific antibody	Blocks EGFR and MET signalling and recruits immune effector cells through ADCC and ADCP.	Activity was modest in unselected GEA [33]. AMIGO, **phase 2**, selected EGFR- or MET-amplified disease and reported disease control in 71% [34].	Infusion reactions, acneiform rash, paronychia, oedema/hypoalbuminaemia and venous thromboembolism.	Investigational.

## Data Availability

No new data were created or analysed in this study. Data sharing is not applicable to this article.

## Abbreviations

ADC	Antibody–drug conjugate
ADCC	Antibody-dependent cellular cytotoxicity
ADCP	Antibody-dependent cellular phagocytosis
BiTE	Bispecific T-cell engager
CAR-T	Chimeric antigen receptor T-cell
CD3	Cluster of differentiation 3
CDC	Complement-dependent cytotoxicity
CI	Confidence interval
CLDN18.2	Claudin 18.2
CPS	Combined positive score
CR	Complete response
CRS	Cytokine release syndrome
ctDNA	Circulating tumour DNA
DCR	Disease control rate
DOR	Duration of response
EGF	Epidermal growth factor
EGFR	Epidermal growth factor receptor
EMA	European Medicines Agency
FDA	Food and Drug Administration
FGFR2b	Fibroblast growth factor receptor 2
G	Gastric
GCN	Gene copy number
GEA	Gastroesophageal adenocarcinoma
GOJ	Gastroesophageal junction
HER2	Human epidermal growth factor receptor 2
HGF	Hepatocyte growth factor
HLH	Haemophagocytic lymphohistiocytosis
HR	Hazard ratio
ICI	Immune checkpoint inhibitor
ICANS	Immune effector cell-associated neurotoxicity syndrome
IHC	Immunohistochemistry
ISH	In situ hybridisation
LVEF	Left ventricular ejection fraction
mAb	Monoclonal antibody
MET	Mesenchymal-epithelial transition factor
MMAE	Monomethyl auristatin E
dMMR	Mismatch repair deficiency
NMPA	National Medical Products Administration
NGS	Next generation sequencing
NSCLC	Non-small cell lung cancer
ORR	Objective response rate
OS	Overall survival
PD-1	Programmed cell death protein 1
PD-L1	Programmed cell death-ligand 1
PFS	Progression-free survival
TAP	Tumour area positivity
TGF-α	Transforming growth factor-α
THBS1	Thrombospondin-1
TKI	Tyrosine kinase inhibitor
TRAE	Treatment-related adverse event
US	United States
VEGF	Vascular endothelial growth factor
VEGFR2	Vascular endothelial growth factor receptor 2
